# Knee Point Search Using Cascading Top-*k* Sorting with Minimized Time Complexity

**DOI:** 10.1155/2013/960348

**Published:** 2013-08-27

**Authors:** Zheng Wang, Shian-Shyong Tseng

**Affiliations:** ^1^Computer Network Information Center, Chinese Academy of Sciences, Beijing 100190, China; ^2^China Organizational Name Administration Center, Beijing 100028, China; ^3^Department of Information Science and Applications, Asia University, Taichung 41354, Taiwan

## Abstract

Anomaly detection systems and many other applications are frequently confronted with the problem of finding the largest knee point in the sorted curve for a set of unsorted points. This paper proposes an efficient knee point search algorithm with minimized time complexity using the cascading top-*k* sorting when a priori probability distribution of the knee point is known. First, a top-*k* sort algorithm is proposed based on a quicksort variation. We divide the knee point search problem into multiple steps. And in each step an optimization problem of the selection number *k* is solved, where the objective function is defined as the expected time cost. Because the expected time cost in one step is dependent on that of the afterwards steps, we simplify the optimization problem by minimizing the maximum expected time cost. The posterior probability of the largest knee point distribution and the other parameters are updated before solving the optimization problem in each step. An example of source detection of DNS DoS flooding attacks is provided to illustrate the applications of the proposed algorithm.

## 1. Introduction

Anomaly detection system and many other applications often rely on finding the largest knee point in the sorted curve to perform clustering, classification, anomaly identification, and so forth [1–6]. Here the largest knee point is targeted because the particular interests lie in finding the cluster of the largest points whose values differ significantly from their lower neighbors in the sorted curve. 

Knee point is defined as the point whose value is close to its upper neighbor while far from its lower neighbor in the sorted curve and thereby taken as the boundary of the cluster of upper points. For an unsorted list, it is necessary to sort it to facilitate the knee point search. Due to time and space efficiency considerations, the method of first completely sorting and then searching the sorted list is often not the optimal one. An alternative approach is to perform search on the partially sorted, namely, top-*k*, list, hoping to save the cost of sort. Therefore the top-*k* sort algorithm is introduced to help minimize the time complexity of the knee point search in this paper. There have been many efforts for bounding and evaluating the time and space complexity of sort algorithms [7–12]. These works provide component algorithms for our work. But the problem of knee point search via top-*k* sorting has not been addressed by any of the previous works. We present in this paper a knee point search algorithm using top-*k* sorting with minimized time complexity. 

This paper is organized as follows: some basic concepts and definitions on knee point search and top-*k* sorting are presented in Section 2; Section 3 will design a knee point search algorithm, including basic idea, top-*k* sort algorithm, time complexity, parameter updating, cascading top-*k* sorting with minimized time complexity, the knee point search algorithm, and the solution of the optimization problem; Section 4 will introduce source detection of DNS DoS flooding attacks as an application example of the proposed algorithm; Section 5 will conclude this paper. 

## 2. Knee Point Search and Selection Sort

Assume there are *n* points *a*_1_, *a*_2_, …, *a*_*n*_ whose values are *q*_1_, *q*_2_, …, *q*_*n*_. Let *a*_1_, *a*_2_, …, *a*_*n*_ be sorted by their values and let their differential values be *δ*_1_, *δ*_2_, …, *δ*_*n*_, where *q*_1_ ≥ *q*_2_ ≥ ⋯≥*q*_*n*_, and

(1) $$\begin{array}{c} \delta_{i}=\{\begin{array}{cc} \delta_{1} & i=1\\ q_{i-1}-q_{i} & i=2,3,\ldots ,n. \end{array} \end{array}$$

**Figure 1:**
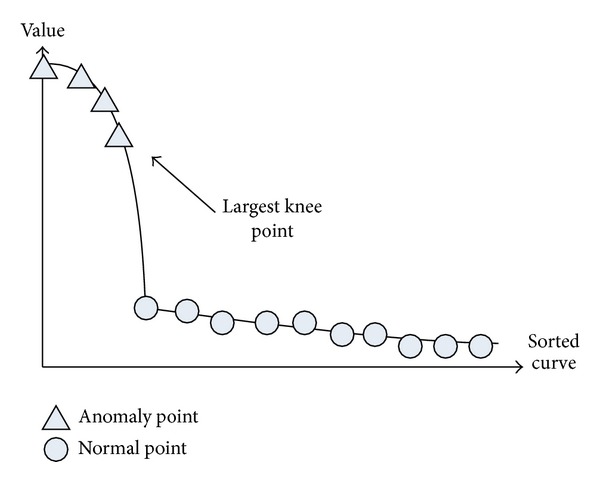
Sorted graph for first knee point search.

As illustrated in Figure 1, there is usually a notable gap of value between points on the upper left side and those on the lower right side of the sorted curve. We define a knee point as the one whose neighboring differential values differ significantly.

**Definition 1**

Point  *a*_*i*_, *i* = 1, 2, …, *n* − 1, is a knee point if it satisfies

(2) $$\begin{array}{c} \frac{\delta_{i+1}}{\delta_{i}}>\theta , \end{array}$$

where *θ* is the threshold, whose value ranges from 10 to 50 in the practice of anomaly detection.

Note that there may be more than one knee point for a list, and the goal of the algorithm is to find the largest one in the sorted curve. For an unsorted list, we should first partially sort the list to find the sorted top-*k *list and then search the sorted top-*k *list for the largest knee point.

**Definition 2**

A top-*k* sort problem of selection number *k *for an unsorted list *L* is a problem that finds *k* largest elements of *L* sorted in descending order.

Apparently, total sort is often not optimal for the problem as knee point search may be successful on a partially sorted top-*k* list if it contains the largest knee point. Therefore it is preferable to selectively sort first using the top-*k* sort algorithm and then search in each step. The procedure may go through many recursive steps until finding the largest knee point for the search may fail in the previous steps. There is a tradeoff between the time cost and expected hit probability of knee point search in each step, both determined by the selection number and both contributing to the expected overall time cost. In this paper, the optimization problem of the selection number is solved by minimizing the maximum expected time cost. 

## 3. The Knee Point Search Algorithm

### 3.1. Basic Idea

The knee point search algorithm is based on cascading top-*k* sorting. In each step, top-*k* sorting segments the list left to be searched. The optimal selection number is determined by minimizing the expected time cost on the list left. If the search successfully finds the knee point in the sorted top-*k* list, the algorithm ends there. Otherwise, the residual list excluding top-*k* requires further checking. It becomes the objective list for the next step and the function of the expected time cost using top-*k *sorting should also be updated according to the a priori knowledge that the search fails in the previous step. Thus the new top-*k* sort problem, likewise, holds for the next step. The algorithm runs in this way recursively until the knee point is found. There are two cases that bring the algorithm to the end.

1. The knee point is found in the sorted top-*k* list in a step.
2. The optimal selection number in one step equals the length of the objective list. This means the optimal option is total sorting. Therefore the knee point is certain to be found in the completely sorted list.

### 3.2. Top-*k* Sort Algorithm

We design a quicksort variation as the top-*k* sort algorithm. Quicksort is a very efficient sort algorithm invented by Hoare [7]. Quicksort has two phases: the partition phase and the sort phase, which makes it a good example of the divide and conquer strategy for solving problems. 

Top-*k* sorting only aims at treating the largest *k* elements, and thus it can be facilitated by the divide and conquer strategy. The intermediate results of quicksort, namely, the pivot positions, can be leveraged to possibly cut off one of the smaller problems divided from the bigger problem and to be conquered. For the strategy to be effective, the partition phase runs recursively only for the lower part if the pivot falls below position *k*, because there is no need to sort the upper part, which only consists of elements larger than top-*k*. This is the major distinction from the original quicksort algorithm and brings sorting efficiency. 

At the same time, the pivots located after position *d*_*i*_ (the optimal selection number) at step *i* in Section 3.6 are potentially useful for the afterwards steps, while they are actually not helpful for the inner top-*d*_*i*_ sorting. Therefore we record those pivots via a stack. A stack is a data structure featured by last in, first out (LIFO). Recalling that the recursive partitions with their pivots after position *k* produce their pivots in a sequential descending position order, we push these pivots into the stack resulting in a stack of pivots ordered by their positions. At the afterwards step *i* + 1, if the optimal selection number *d*_*i*+1_ is larger than the position of the pivot at the top of the stack, the pivot is popped from the stack used for an inner pivot of top-*d*_*i*+1_. Since this pivot is no longer needed for the afterwards steps, it should not be maintained in the stack. When the stack is empty or the pivot at the top of the stack (so do all of the other pivots) is located after the selection number, the partition has to run by itself to find a pivot without the help of the pivot stack. 

The top-*k* sort algorithm, namely, QuickSortTopK, can be expressed as in Algorithm 1.

The input of QuickSortTopK is the objective unsorted list *L* indexed from FirstIndex to LastIndex. For all steps, LastIndex is fixed at *n*, whereas FirstIndex is progressively increased to exclude the sorted part of *L* in all previous steps. The output includes the sorted top-*k* elements of *L* indexed from FirstIndex to LastIndex and the stack *S* containing all pivots falling after position *k* obtained in all previous steps. 

The termination condition of the recursion is checked in Line 1 of the algorithm. If the stack is nonempty and the top element of the stack falls into the objective range (see Line 2), the top element is used as the pivot for the partition (see Line 3). Otherwise, the pivot is obtained by a partition (see Line 6). Once the pivot is presented, different recursive steps are to be taken depending on the position of the pivot. If the pivot falls after position *k*, it should be pushed into the stack and then run further sorting on the original list subtracting the pivot, hoping to help afterwards steps (see Lines 9, 11). If the pivot is located exactly at position *k*, the pivot itself is the last element of the output and thereafter only top-*k*-1 sorting on the original list subtracting the pivot is needed (see Line 15). If the pivot is located prior to position *k*, both the upper and lower parts should be treated. The action on the upper part is equivalent to Quicksort, while the action on the lower part is actually the recursive running of QuickSortTopK with diminished selection number *k* and the shrinking objective list (see Lines 18, 20). 

### 3.3. Time Complexity

Let totally sorting of a list of length *n* require *S*(*n*) time. Calculated by the number of comparisons, the average time complexity of *S*(*n*) is *O*(*n*log⁡(*n*)) following some efficient algorithms, for example, Quicksort [7]. 

Let top-*k* sorting of selection number *k* require *C*(*k*, *n*) time, where *n* is the length of the list. The QuickSortTopK algorithm requires an expected time of *O*(*n* + *k*log⁡*k*). So *C*(*k*, *n*) equals *O*(*n* + *k*log⁡*k*). 

Let the time complexity of finding the knee point in the sorted list of length *n* be *D*(*n*). Recalling (2), *D*(*n*) takes *O*(*n*). 

### 3.4. Parameter Updating

For a list of length *n*, the algorithm divides the overall procedure into *m* + 1 steps by a sequence of selection numbers, *d*_1_, *d*_2_,…, *d*_*m*_, 1 ≤ *d*_1_ < *d*_2_ < ⋯<*d*_*m*_ ≤ *n*. Additionally, to facilitate the formulation, we let *d*_0_ = 0 and *d*_*m*_ + 1 = *n*. Let the length of the objective list in step *i*, *i* = 1,2,…, *m* + 1 be *n*_*i*_; we have

(3) $$\begin{array}{c} n_{i}=n-d_{i-1},\;i=\;1,2,\ldots ,m+1. \end{array}$$

In the first step, *n*_*i*_ = *n*. Let top-*k*_*i*_ sorting for the objective list of length *n*_*i*_ be performed in step *i*, *i* = 1,2,…, *m* + 1, and we have

(4) $$\begin{array}{c} k_{i}=d_{i}-d_{i-1},\;i=\;1,2,\ldots ,m+1. \end{array}$$

Particularly, *k*_*i*_ = *n*_*i*_ in step *m* + 1. And thus top-*k*_*i*_ sorting for the objective list of length *n*_*i*_ is actually total sorting of the objective list. If the search of the knee point is successful in step *i* for the sorted top-*k*_*i*_ list, the algorithm ends at step *i*. Otherwise, the algorithm continues with the next step. The algorithm lasts until step *m* + 1 if the search misses in all of the previous steps during the progressive search. Since step *m* + 1 takes no further selection of the objective list, the algorithm finishes in it. 

Let *A* be the position variable of the knee point and *A* = 1, 2,…, *n*. Let *p*(*i*) represent the probability that *A* = *i*, *i* = 1, 2,…, *n*, and thus ∑_*i*=1_^*n*^ *p*(*i*) = 1. The value of *p*(*i*) is assumed to be known at the beginning of the algorithm. Let *A*_*i*_ be the position variable of the knee point in step *i* and *A*_*i*_ = *d*_*i*−1_ + 1, *d*_*i*−1_ + 2,…, *n*. Let *p*_*i*_(*j*) represent the probability that *A*_*i*_ = *j*, *j* = *d*_*i*−1_ + 1, *d*_*i*−1_ + 2,…, *n*, *i* = 1, 2,…, *m* + 1, and thus ∑_*j*=*d*_*i*−1_+1_^*n*^ *p*_*i*_(*j*) = 1.

**Lemma 3**

The probability distribution of the knee point in step *i* *p*_*i*_(*j*) can be written as

(5) $$\begin{array}{c} p_{i}\left(j\right)=\frac{p\left(j\right)}{\sum_{k=d_{i-1}+1}^{n}p\left(k\right)},\\ j=d_{i-1}+1,\;\;d_{i-1}+2,\ldots ,n,\;i=1,2,\ldots ,m+1. \end{array}$$

**Proof**

At the first step, all knowledge about the probability distribution of the knee point is only given by *p*(*i*). But the search in the afterward steps should make use of the posterior distribution of the knee point for it is confirmed not to exist prior to the selection number in the previous steps; for example, when the algorithm comes to step *i*, the knee point is already checked to be not present in the top-*d*_*i*−1_ list, *i* = 2, 3,…, *m* + 1. Therefore *p*_*i*_(*j*) should be updated in step *i* as

(6) pi(j)=P(Ai=j)=P(A=j ∣ A∈{di−1+1,di−1+2,…,n})=P(A=j,j∈{di−1+1,di−1+2,…,n})P(A∈{di−1+1,di−1+2,…,n})=p(j)∑k=di−1+1np(k)j  =  di−1+1,  di−1+2,…,n, i  =  1,2,…,m+1.

Let the hit probability of search in step *i* be *H*_*i*_.

**Lemma 4**

The hit probability of search in step *i* *H*_*i*_ can be written as

(7) $$\begin{array}{c} H_{i}\left(d_{i}\right)=\sum_{j=d_{i-1}+1}^{d_{i}}p_{i}\left(j\right),\;i=1,2,\ldots ,m+1. \end{array}$$

**Proof**

For the selection number *d*_*i*_ in step *i*, the search for the knee point is successful if and only if the knee point falls into the interval of position among *d*_*i*−1_ + 1 and *d*_*i*_. According to Lemma 3, the probability distribution of the knee point at step *i* *p*_*i*_(*j*) should be updated as (5). Therefore we have

(8) Hi(di)=∑j=di−1+1diP(Ai=j)=∑j=di−1+1dipi(j), i=1,2,…,m+1.

### 3.5. Cascading Top-*k* Sorting with Minimized Time Complexity

**Lemma 5**

Let the expected overall computational time cost in step *i* be *tc*_*i*_; *tc*_*i*_ yields

(9) tci=C(di−di−1,n−di−1) +(1−Hi(di))tci+1, i  =  1,2,…,m.

**Proof**

When the search succeeds in step *i*, tc_*i*_ comes only from top-*k*_*i*_ sorting which requires *C*(*k*_*i*_, *n*_*i*_) time and the search in the top-*k*_*i*_ sorted list which requires *D*(*n*). However, recalling Section 3.3, the time complexity of *D*(*n*) is negligible compared to that of *C*(*k*_*i*_, *n*_*i*_), so the summation of them can be approximated by *C*(*k*_*i*_, *n*_*i*_). For the failure of search in step *i*, the residual list of length *n*_*i*_ − *d*_*i*_ has to be further checked. Thus the overall computation time cost consists of top-*k* sort and search in the remaining list. Note that the computation time cost of sort and search in the remaining list is no other than tc_*i*+1_. Hence tc_*i*_ yields

(10) tci=Hi(di)C(ki,ni)+(1−Hi(di))(C(ki,ni)+tci+1)=C(ki,ni)+(1−Hi(di))tci+1,   i  =  1,2,…,m.

Plugging (3) and (4) into (10), we get (9).

Lemma 5 tells us that the expected computational cost tc_1_ can be calculated iteratively following (7), until reaching tc_*m*+1_ where there is no selection for step *m* + 1. Thus tc_*m*+1_ only consists of the time cost of total sorting of the list of length *n*_*i*_ and the search in it. As total sorting takes *S*(*n*_*i*_) and search takes *D*(*n*_*i*_), thus we have

(11) $$\begin{array}{c} {\text{tc}}_{m+1}=S\left(n_{m+1}\right)+D\left(n_{m+1}\right). \end{array}$$

Let *N*(*a*, *b*) (*a* ≤ *b* and *a*, *b* are integers) denote the set of integers {*i* | *a* ≤ *i* ≤ *b*  and  *i*  is  integer}. In every step *i*, the algorithm calculates the current probability distribution of the knee point which determines the hit probability *H*_*i*_ and chooses *d*_*i*_ to be the solution of the following optimization problem:

(12) Min⁡: tci s.t.     di∈N(di−1+1,n).

We see in (12) that for any fixed *d*_*i*_ the minimum of tc_*i*_ is determined by tc_*i*+1_ under the optimal selection of *d*_*i*+1_ in the next step, *d*_*i*_ ∈ *N*(*d*_*i*−1_ + 1, *n*). And the optimal tc_*i*+1_ for any fixed *d*_*i*+1_ is also determined by the tc_*i*+2_ under the optimal selection of *d*_*i*+2_ and so on. This kind of iterative dependency finally extends to the last step which has no further selection. So tc_*i*_ is the function of *d*_*i*_, *d*_*i*+1_,…. The variation of any choice of selection in any number of steps makes the search space of optimization very huge, especially for the initial steps. Therefore it is not practical to evaluate all possibilities of selections in all of the afterwards steps when solving the optimization problem in step *i*. Thus it is necessary to constrain the variable of the objective function tc_*i*_ in (12) as mere *d*_*i*_.

**Theorem 6**

The upper bound of the minimum of *tc*_*i*_ for a fixed *d*_*i*_ can be written as substituting *tc*_*i*+1_ in (9) by *S*(*n*_*i*+1_) + *D*(*n*_*i*+1_), such that

(13) min⁡di+1∈N(di+1,n),di+2∈N(di+1+1,n),…⁡tci ≤C(di−di−1,n−di−1)  +(1−Hi(di))(S(n−di)+D(n−di)),               i  =  1,2,…,m.

**Proof**

We only need to prove that

(14) $$\begin{array}{c} \underset{d_{i+1}\in N\left(d_{i}+1,n\right),d_{i+2}\in N\left(d_{i+1}+1,n\right),\ldots }{\min}{\text{tc}}_{i+1}\leq S\left(n-d_{i}\right)+D\left(n-d_{i}\right). \end{array}$$

As total sort of the list of length *n*_*i*+1_ can be viewed as performing top-*n*_*i*+1_ sorting, their time costs are both *S*(*n*_*i*+1_) + *D*(*n*_*i*+1_). This is equivalent to the case when *d*_*i*+1_ = *n*. Because the algorithm ends with the total sorting and there are no afterwards steps, we have no definitions for *d*_*i*+1_, *d*_*i*+2_,…. Thus we have

(15) $$\begin{array}{c} {{\text{tc}}_{i+1}|}_{d_{i+1}=n\;\;(\text{hence}\;\;d_{i+2},d_{i+3\ldots }\;\;\text{has}\;\;\text{no}\;\;\text{definition})}=S\left(n_{i+1}\right)+D\left(n_{i+1}\right). \end{array}$$

Hence

(16) min⁡di+1∈N(di+1,n),di+2∈N(di+1+1,n),…tci+1 ≤tci+1|di+1=n  (hence  di+2,di+3,…has  no  definition).

Plugging (3) into (15) and then (15) into (16), we have (14), and thereby (13) is proved.

Theorem 6 manifests that, for a fixed *d*_*i*_, tc_*i*_ is definitely bounded by the time cost of total sort of the residual list of length *n*_*i*_ + 1  plus that of search in it. The optimization problem in step *i* described by (13) can be isolated from all of the possible selections of the afterwards steps and becomes a function of mere *d*_*i*_. This minmax technique brings convenience to our analysis, such that (13) can be simplified as

(17) Min⁡: C(di−di−1,n−di−1)  +(1−Hi(di))(S(n−di)+D(n−di)) s.t.    di∈N(di−1+1,n).

Plugging (5) into (17), we have

(18) Min⁡: C(di−di−1,n−di−1) +(1−∑j=di−1+1dipi(j))(S(n−di)+D(n−di)) s.t.    di∈N(di−1+1,n).

Solving (14), we can obtain the optimal *d*_*i*_ in step *i*.

### 3.6. The Knee Point Search Algorithm

The knee point search algorithm runs iteratively using cascading top-*k* sorting. When the optimal selection number *d*_*i*_ is determined at step *i*, top-*k* sorting can be done via running QuickSortTopK  (*L*, *d*_*i*−1_ + 1, *n*, *d*_*i*_ − *d*_*i*−1_).  Specifically, the first step starts with QuickSortTopK (*L*, 1, *n*, *d*_1_). 

The procedure can be described as follows.

**Step 1**

  (1) According to (5), the probability distribution of the knee point for the optimization problem is initialized as follows
  (19) $$\begin{array}{c} p_{1}\left(j\right)=p\left(j\right),\;j\;=\;1,2,\ldots ,n. \end{array}$$
(2)  The optimal selection number *d*_1_ is obtained by solving the following optimization problem as (18):
  (20) Min⁡: C(d1,n)+(1−∑j=1d1p(j))   ×(S(n−d1)+D(n−d1)) s.t.   d1∈N(1,n).
(3)  Perform *top*-*d*_1_ sorting on the list of length *n*.
(4)  Search for the knee point on the sorted top-*d*_1_ list. If successful or *d*_1_ = *n*, the algorithm ends. Otherwise, go to Step 2.

**Step 2**

(1)  The probability distribution of the knee point for the optimization problem is updated as follows:
  (21) $$\begin{array}{c} p_{2}\left(j\right)=\frac{p\left(j\right)}{\sum_{k=d_{1}+1}^{n}p\left(k\right)},\;j=d_{1}+1,\;\;d_{1}+2,\ldots ,n. \end{array}$$
(2) *d*_2_ is derived as the solution of the following optimization problem:
  (22) Min⁡: C(d2−d1,n−d1)+(1−∑j=d1+1d2p2(j))   ×(S(n−d2)+D(n−d2))s.t.    d2∈N(d1+1,n).

Note that *d*_1_ is inherited from Step 1.

(3) Perform top-(*d*_2_ − *d*_1_) selection sort on the list of length *n* − *d*_1_ subtracting top-*d*_1_ elements in Step 1.
(4) Search for the knee point on the sorted top-(*d*_2_ − *d*_1_) list. If successful or *d*_2_ = *n*, the algorithm ends. Otherwise, go to Step 2.

*Step i*.

1. The probability distribution of the knee point for the optimization problem is updated according to (5).
2. *d*_*i*_ is obtained as the solution of the optimization problem in (18), where *d*_*i*−1_ is known from step *i* − 1.
3. Perform top-(*d*_*i*_ − *d*_*i*−1_) selection sort on the list of length *n* − *d*_*i*−1_ subtracting top-*d*_*i*−1_ elements already sorted in the previous steps.
4. Search for the knee point on the sorted top-(*d*_*i*_ − *d*_*i*−1_) list. If successful or *d*_*i*_ = *n*, the algorithm ends. Otherwise, go to step *i* + 1.

The knee point search algorithm can be summarized as in Algorithm 2.

The knee point search algorithm can also be expressed recursively. 

For each recursive step, we have an unsorted list *L* of length *n* and the probability distribution of the knee point in the sorted list of length *n*:  *P*. Thus we can modify the optimization problem in (18) as follows:

(23) Min⁡: C(d,n)+(1−∑j=1dp(j))(S(n−d)+D(n−d)) s.t.  d∈N(1,n).

Solving (14), we can obtain the optimal *d* in each recursive step. 

When the knee point search fails after top-*k* sorting in each recursive step, the algorithm has to go to the next recursive step. First, we need to update the probability distribution of the knee point as well as the residual unsorted list as two parameters for the recursive function. According to Lemma 3, the update of the probability distribution of the knee point yields

(24) $$\begin{array}{c} p\left(j\right)⟵\frac{p\left(j\right)}{\sum_{k=d+1}^{n}p\left(k\right)},\;j=d+1,\;\;d+2,\ldots ,n. \end{array}$$

In each recursive step, top-*k* sorting can be done via running QuickSortTopK(*L*, 1, length(*L*), *d*), where *L* is the objective list in the current step and *length *(*L*) denotes the length of  *L*. 

A recursive version of knee point search algorithm can be summarized as in Algorithm 3.

### 3.7. The Solution of the Optimization Problem

In this section, we will assume two forms of the probability distribution of the knee point and discuss the solutions of (18) under these presumptions.

*(1) Uniform Distribution*. *p*(*i*) is equal for all *i* ∈ *N*(1, *n*), and thus *p*(*i*) = 1/*n*. Plugging *p*(*i*) into (23), we have

(25) Min⁡: C(d,n)+(1−dn)(S(n−d)+D(n−d)) s.t.     d∈N(1,n).

Let

(26) $$\begin{array}{c} f\left(d\right)=C\left(d,n\right)+\left(1-\frac{d}{n}\right)\left(S\left(n-d\right)+D\left(n-d\right)\right). \end{array}$$

Although (26) is a discrete function, we still utilize the method of derivation to find the extremum, which can be only applied to the continuous and derivable function. Here we treat the discrete variable *d* as continuous ones, and thereafter (26) turns into a continuous and derivable function. This is a rational approximation of the problem, which facilitates our analysis and solving. The final solution should be the round-off of *d* obtained by solving the continuous function.

For simplicity, we let

(27) $$\begin{array}{c} C\left(k,n\right)=c_{1}\left(n+k\log k\right)\\ S\left(n\right)=c_{2}\left(n\log\left(n\right)\right)\\ D\left(n\right)=c_{3}n, \end{array}$$

where *c*_1_,  *c*_2_, and *c*_3_ are all constants.

By choosing *d* such that *df*(*d*)/*d* = 0, we have the optimal *d* which satisfies

(28) $$\begin{array}{c} c_{1}\left(1+\log d\right)n=\left(n-d\right)\left[2c_{2}\log\left(n-d\right)+2c_{3}+c_{2}\right]. \end{array}$$

For large *n* and *d*, we have the approximation of (28) as

(29) $$\begin{array}{c} c_{1}n\log d=2c_{2}\left(n-d\right)\log\left(n-d\right). \end{array}$$

To get the explicit mathematical expression of the solution of the nonlinearity equation (29), we used a heuristic approach to simplify the problem. We assume that *d* is a proportion function of *n*, such that

(30) $$\begin{array}{c} d=pn,\;0<p<1, \end{array}$$

where *p* decides the optimum of *d*.

Thus (30) yields

(31) $$\begin{array}{c} c_{1}n\log\left(pn\right)=2c_{2}\left(1-p\right)n\log\left[\left(1-p\right)n\right], \end{array}$$

then

(32) c1nlog⁡⁡(p)+c1nlog⁡⁡(n) =2c2(1−p)nlog⁡⁡(1−p)+2c2(1−p)nlog⁡⁡(n).

Leave out the low order item in both sides of (32), and we get

(33) c1nlog⁡⁡(p)+c1nlog⁡⁡(n)≈c1nlog⁡⁡(n),2c2(1−p)nlog⁡⁡(1−p)+2c2(1−p)nlog⁡⁡(n)  ≈2c2(1−p)nlog⁡⁡(n).

Plugging (33) into (32), the optimum of *p* yields

(34) $$\begin{array}{c} p=1-\frac{c_{1}}{2c_{2}}. \end{array}$$

We can see that the optimum of *p* that takes the form of a proportion function of *n* exists only when *c*_1_ < 2*c*_2_. Particularly, if *c*_1_ = *c*_2_ = 1, the optimum of *p* is 1/2, which means that the optimum first selection number is half of the length of the list. As the search algorithm may run recursively if the knee point search misses, the optimal method is the so-called binary search or logarithmic search method.

**Theorem 7**

If the probability distribution of the knee point follows uniform distribution and the optimal selection number takes the form of (30), the optimal method for the knee point search algorithm is binary search or logarithmic search method.

**Proof**

We first prove using an inductive method that in each recursive step of the search algorithm, *p*(*i*) is equal for all *i* ∈ *N*(1, *n*).

We know that for the first recursive step, *p*(*i*) is equal for all *i* ∈ *N*(1, *n*) as the starting point of the induction. Then for the second recursive step, we can derive according to (24) that *p*(*i*) is equal for all *i* ∈ *N*(1, *n*).

Suppose that for the *l*th recursive step, *p*(*i*) is equal for all *i* ∈ *N*(1, *n*). Similarly, for the (*l* + 1)th recursive step, *p*(*i*) is equal for all *i* ∈ *N*(1, *n*) following (24).

Therefore we can form the concept inductively that in each recursive step of the knee point search algorithm, *p*(*i*) is equal for all *i* ∈ *N*(1, *n*). So the optimization problem in each recursive step can be written as no other than (25), whose solution of number of selection is half of the length of the list discussed above. Here the list is the one left from previous recursive step. Hence the optimal method for the search algorithm is binary search or logarithmic search method.

*(2) Inverse Proportion Distribution*. *p*(*i*) is in direct inverse proportion to *i*, *i* ∈ *N*(1, *n*), and thus *p*(*i*) = *c*/*i*, where *c* = 1/∑_*i*=1_^*n*^(1/*i*).

As an approximate treatment of the summation ∑_*i*=*a*_^*b*^(1/*i*), we consider

(35) $$\begin{array}{c} \sum_{i=a}^{b}\frac{1}{i}\approx \int_{a}^{b}\frac{1}{x}dx. \end{array}$$

Particularly, for *a* = 1 and a large *b*, we have

(36) $$\begin{array}{c} \sum_{i=1}^{b}\frac{1}{i}=\ln b+E. \end{array}$$

And for a large *n*, we have

(37) $$\begin{array}{c} c=1/\left(\ln n+E\right), \end{array}$$

where *E* is the Euler constant and has the approximate value as 0.5772. 

Plugging *p*(*i*) and then (36) and (37) into (23), we have

(38) Min⁡: C(d,n)+(1−ln⁡⁡d+Eln⁡⁡n+E)(S(n−d)+D(n−d)) s.t. d∈N(1,n).

Let

(39) $$\begin{array}{c} g\left(d\right)=C\left(d,n\right)+\left(1-\frac{\ln d+E}{\ln n+E}\right)\left(S\left(n-d\right)+D\left(n-d\right)\right). \end{array}$$

**Theorem 8**

If the probability distribution of the knee point *p*(*i*) is in direct inverse proportion to *i*,  *i* ∈ *N*(1, *n*), the optimal top-*k* sorting for the knee point search algorithm is full sorting of the list for a large length of the list.

**Proof**

We only need to prove that *g*(*d*) is a monotony increase function of *d*, and the equivalent condition for it is

(40) dg(d)d=c1(1+log⁡⁡d)−c2(n−d)log⁡⁡(n−d)−c3n(ln⁡⁡n+E)d −c2(ln⁡⁡n−ln⁡⁡d)(1+log⁡⁡(n−d))ln⁡⁡n+E<0,

that is,

(41) c1(1+log⁡⁡d)(ln⁡⁡n+E)d <c2(n−d)log⁡⁡(n−d)  +c3n+c2(ln⁡⁡n−ln⁡⁡d)(1+log⁡⁡(n−d))d.

We assume that *d* is an exponential function of *n*, such that

(42) $$\begin{array}{c} d=n^{q},\;0<q<1. \end{array}$$

Thus the left and right sides of (41) can be, respectively, written as

(43) $$\begin{array}{c} c_{1}\left(1+\log d\right)\left(\ln n+E\right)d\sim O\left({\left(\log n\right)}^{2}n^{q}\right) \end{array}$$

(44) c2(n−d)log⁡⁡(n−d)+c3n +c2(ln⁡⁡n−ln⁡⁡d)(1+log⁡⁡(n−d))d~O(n).

Comparing (43) and (44), we obtain (41) for a large *n*. That means the optimal selection number is *n* for the first step, or the optimal top-*k* sorting for the knee point search algorithm is full sorting.

## 4. Source Detection of DNS DoS Flooding Attacks: An Application Example

### 4.1. DNS DoS Flooding Attacks

The Domain Name System is a fundamental and indispensable component of the modern Internet [13, 14]. The availability of the DNS can affect the availability of a large number of Internet applications. Ensuring the DNS data availability is an essential part of providing a robust Internet. 

In the past few years, some important DNS name servers on the top level of the DNS hierarchical structure were targeted by the DoS or DDoS attackers, and some of these attacks did succeed in disabling the DNS servers and resulted in parts of the Internet experiencing severe name resolution problems [15–18]. Particularly, DNS DoS flooding attacks are the attacks launched by the attackers towards the DNS name servers with an overwhelming traffic flux in order to disrupt the DNS service for the legitimate clients. However, it is usually not easy to efficiently detect and defend the DoS flooding attacks because the attacking traffic is blended with the legitimate ones, which complicates the distinguishing efforts. Moreover, the detection mechanism should be implementable or should not add heavy computational load. Here we focus on the source-based detection method and show that the problem of source detection of DNS DoS flooding attacks can be addressed by the knee point search in the sorted curve discussed in this paper. 

### 4.2. Detection Using the Knee Point Search

Generally, DNS name servers may receive queries coming from thousands of DNS clients (mostly DNS cache servers), whose traffic volumes are expected to remain far below those of the DoS flooding attacks. The real-time query rates for all incoming sources can be counted by the traffic monitoring system residing at the border gateway in front of the DNS name server. The goal of the DoS attack defense is to realize real-time attacking source detection and then filter out the attacking traffic from these sources accordingly. Therefore the detection problem is equivalent to knee point search in the sorted curve, where all points above the largest knee point are identified as the attacking sources. Moreover, time efficiency is also the key requirement for the problem, for timely attacking detection means timely defending action. Applying the knee point search algorithm proposed in this paper, the expected detection time is minimized. 

### 4.3. Leaning the Knee Point Distribution

The assumption on probability distribution of the knee point is the prerequisite for the knee point search algorithm. However, in the initial rounds of detection we have hardly any a priori knowledge about the knee point. But the distribution estimation of the knee point can be learned based on the empirical data obtained in all previous rounds of detection. 

First, suppose that the knee point largely follows stationary random distribution; hence its distribution exhibits almost the same probability model in all rounds of detection. We can fit a statistical model to data and provide estimates for the model's parameters. Here we apply the method of maximum likelihood for the estimation.

Let the count variable of detected knee points so far at position *i* in the ordered list of length *n* be *h*_*i*_,  *i* = 1,2,…, *n*. Let the value of *h*_*i*_ be *x*_*i*_, *i* = 1,2,…, *n*. If the number of rounds of detection is *R*, we have

(45) $$\begin{array}{c} \sum_{i=1}^{n}x_{i}=R. \end{array}$$

Let the probability vector of the knee point at different positions be *P* = (*p*_1_,*p*_2_,…,*p*_*n*_)′. The likelihood function of *P* can be written as

(46) $$\begin{array}{c} \text{lik}\;\left(P\right)=f_{D}\left(h_{1}=x_{1},h_{2}=x_{2},\ldots ,h_{n}=x_{n}\;|\;P\right), \end{array}$$

where *f*_*D*_(·) is the density function. To calculate lik (*P*), we have

(47) lik(P)=fD(h1=x1 ∣ P)fD(h2=x2,…,hn=xn ∣ h1=x1,P)=fD(h1=x1 ∣ P)fD(h2=x2 ∣ h1=x1,P)fD ×(h3=x3,…,hn=xn ∣ h1=x1,h2=x2,P)⋮=fD(h1=x1 ∣ P)fD(h2=x2 ∣ h1=x1,P)⋯ fD(hn−2=xn−2 ∣ h1=x1,h2=x2,…,hn−3=xn−3,P) ×fD(hn−1=xn−1,hn=xn ∣ h1=x1,     h2=x2,…,hn−2=xn−2,P)=fD(h1=x1 ∣ P)fD(h2=x2 ∣ h1=x1,P)⋯ fD(hn−2=xn−2 ∣ h1=x1, h2=x2,…,hn−3=xn−3,P) ×fD(hn−1=xn−1 ∣ h1=x1,       h2=x2,…,hn−2=xn−2,P) ×fD(hn=xn ∣ h1=x1,h2=x2,…,hn−1=xn−1,P),

where the last item in (47) is actually not an independent one given that all *h*_*i*_ other than *h*_*n*_ are known due to the constraint in (45), such that

(48) $$\begin{array}{c} f_{D}\left(h_{n}=x_{n}\;|\;h_{1}=x_{1},h_{2}=x_{2},\ldots ,h_{n-1}=x_{n-1},P\right)=1. \end{array}$$

Plugging (48) into (47), we get

(49) lik (P)=(nx1)p1x1(1−p1)n−x1(n−x1x2)(p21−p1)x2 ×(1−p21−p1)n−x1−x2⋯(n−∑i=1n−2xixn−1) ×(pn−11−∑i=1n−2pi)xn−1(1−pn−11−∑i=1n−2pi)n−∑i=1n−1xi.

Thus let

(50) $$\begin{array}{c} \frac{\partial \ln\left(\text{lik}\left(P\right)\right)}{\partial p_{i}}=0,\;i=1,2,\ldots ,n-1. \end{array}$$

We obtain the maximum likelihood estimation of *p*_*i*_, *i* = 1,2,…, *n* − 1:

(51) $$\begin{array}{c} {\hat{p}}_{i}=\frac{x_{i}}{R},\;i=1,2,\ldots ,n-1,\\ {\hat{p}}_{n}=1-\sum_{i=1}^{n-1}{\hat{p}}_{i}=\frac{x_{n}}{R}. \end{array}$$

At the beginning of each round of detection, if the previous round finds the knee point at position i*, *R* and *x*_*i*_, *i* = 1,2,…, *n*, are updated as follows:

(52) $$\begin{array}{c} R⟵R+1,\\ x^{i*}⟵x^{i*}+1,\\ x^{i}⟵x^{i},\;i\in N\left(1,n\right),\;\;i\neq i^{*}. \end{array}$$

The knee point distribution may evolve over time; thus the position of the knee point detected in recent rounds provides more reliable information for the estimation than earlier rounds. Taking the chronological order into consideration, we assign more weight to recent rounds than earlier rounds. This can be done by decreasing the detection results in previous rounds progressively. The deceasing is performed in updating *R* and *x*_*i*_, *i* = 1,2,…, *n*, and sums up the current detection and the previous ones at a discount *β*, 0 < *β* < 1. Formally, the updating of *R* and *x*_*i*_, *i* = 1,2,…, *n*, can be modified as follows:

(53) $$\begin{array}{c} R⟵\beta^{*}R+1,\\ x^{i*}⟵\beta^{*}x^{i*}+1,\\ x^{i}⟵\beta *x^{i},\;i\in N\left(1,n\right),\;\;i\neq i^{*}. \end{array}$$

## 5. Conclusion

Knee point search in the sorted curve is often used in the practice of anomaly detection and many other applications. Due to the inefficiency of total sorting, top-*k* sorting should be adopted for the knee point search. In this paper, a knee point search algorithm using cascading top-*k* sorting is proposed. The expected time complexity is minimized via optimizing the selection number *k* in each step. 

## Figures and Tables

**Algorithm 1:**
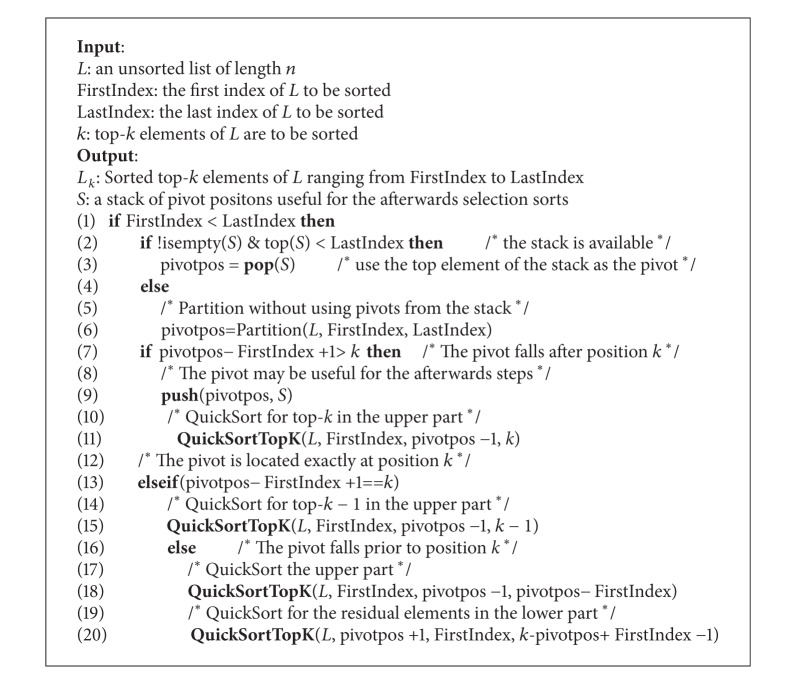
QuickSortTopK(*L*, FirstIndex, LastIndex, *k*).

**Algorithm 2:**
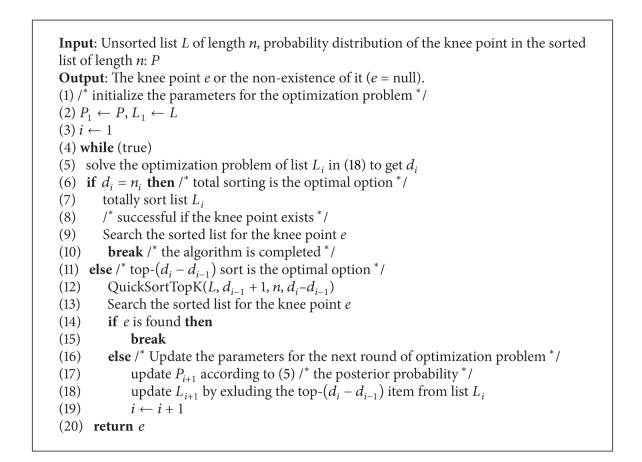
FindKnee(*L*, *P*).

**Algorithm 3:**
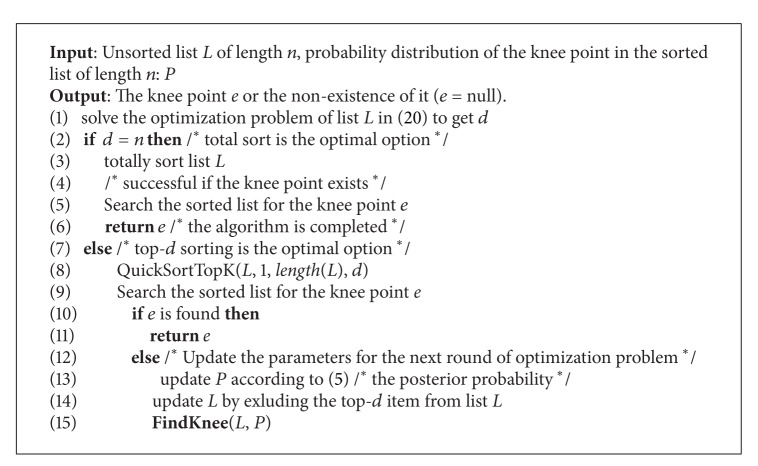
FindKnee(*L*, *P*).
